# Initial fixation of metallic wedge-augmented versus angled BIO reverse shoulder arthroplasty techniques: A finite element study

**DOI:** 10.1177/09544119251356213

**Published:** 2025-07-24

**Authors:** Mariana Lopes, Carlos Quental, Marco Sarmento, João Folgado

**Affiliations:** 1 IDMEC, Instituto Superior Técnico, Universidade de Lisboa, Lisbon, Portugal; 2 Hospital CUF Descobertas, Lisbon, Portugal

**Keywords:** Glenoid bone defect, glenoid retroversion, finite element analysis, wedge-augmented baseplate, bone grafting, micromotions

## Abstract

Augmented techniques in the reverse total shoulder arthroplasty (rTSA) have emerged to treat large asymmetric glenoid bone defects and restore shoulder function. However, whether metallic wedge-augmented (W-AUG-rTSA) and angled bony increased-offset (angled BIO-rTSA) rTSA techniques provide equivalent implant fixation remains unclear. This study aimed to directly compare the initial fixation of W-AUG-rTSA and angled BIO-rTSA in a 15° retroverted glenoid, while also assessing the impact of graft stiffness and the number of peripheral screws. Finite element models were developed considering compressive and inferior-to-superior shear loads. Micromotions at the bone-implant interface were compared between the techniques, considering variations in the number of peripheral screws (2 vs 4) and graft stiffness in the angled BIO-rTSA (96 MPa and 1.3 GPa to simulate different bone qualities, and 2.5 GPa to simulate a porous metal wedge as used in the W-AUG-rTSA). The W-AUG-rTSA and angled BIO-rTSA achieved, respectively, maximum micromotions of 63.5 µm and 47.4–65.0 µm (depending on graft stiffness). Assuming a bone ingrowth threshold of 50 µm, 9% and 0%–4% of the bone-implant interface exceeded this threshold for the W-AUG-rTSA and angled BIO-rTSA techniques, respectively, when 4 peripheral screws were used. These results suggest that both augmentation techniques can likely achieve good initial fixation under this screw configuration. Although changes in graft stiffness affected the micromotion distribution in the angled BIO-rTSA, their overall impact on initial fixation was limited. Reducing the number of peripheral screws to 2 resulted in a substantial increase in interface nodes exceeding the 50 µm threshold in both techniques.

## Graphical abstract

**Graphical abstract:**
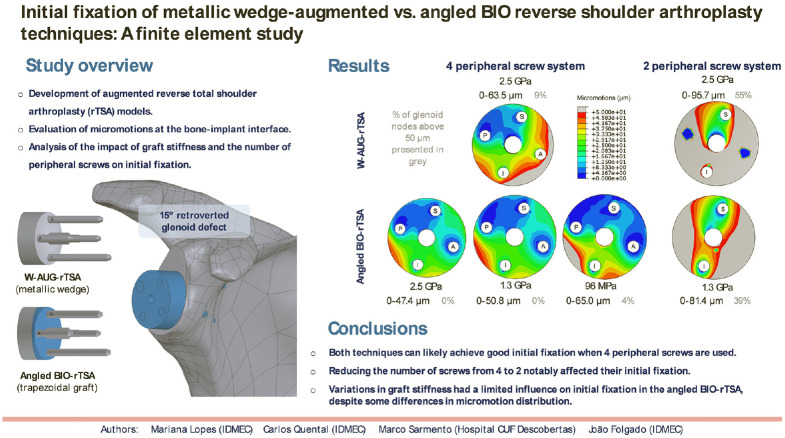

## Introduction

Approximately 40% of patients undergoing primary reverse Total Shoulder Arthroplasty (rTSA) exhibit glenoid deformities and erosion (commonly located posteriorly), increased retroversion, and posterior subluxation of the humeral head.^1–3^ The rTSA procedure is particularly challenging in these patients, who present limited bone stock, often associated with poor bone quality. Under these conditions, surgeons face increased challenges in achieving proper implant placement, potentially contributing to prosthetic stability vulnerability.^1,3,4^ Moreover, severe bone loss may result in excessive medialization of the center of rotation, which contributes to decreased deltoid wrapping around the greater tuberosity, muscle strength, and range of motion, favoring scapular notching.^5–8^ Therefore, correcting glenoid bone loss and deformities in rTSA is crucial to enhance prosthetic stability and longevity.

Current surgical techniques to reconstruct large glenoid bone defects and retroversion angles exceeding 10° include combining eccentric or off-axis reaming with augmented techniques that either use bone grafts with non-augmented baseplates (BIO-rTSA) or wedge-augmented metallic glenoid baseplates (W-AUG-rTSA). These two augmented techniques have emerged to enhance implant fixation; preserve bone stock; and restore joint line and rotator cuff tension by avoiding excessive medialization.^1,2,5,9^ On one hand, bone grafting has become a common option to address severe bone loss. Depending on the defect, the bone graft may be symmetrical (BIO-rTSA) or asymmetrical (angled BIO-rTSA). The angled BIO-rTSA technique consists of harvesting an autologous humeral head graft with a trapezoidal shape and combining it with a non-augmented baseplate to match the glenoid version.^5^ On the other hand, interest in W-AUG-rTSA for addressing asymmetric glenoid wear patterns has been increasing in recent years.^2^ These augmented implants exist in different geometries, depending on the severity of glenoid wear and deformity, and can contain porous titanium or bioactive surface coatings on the baseplate backside to promote bone ingrowth and enhance biological fixation.^6,10^

Despite the W-AUG-rTSA^2,3^ and BIO-rTSA^5,9,11,12^ presenting encouraging functional results in various clinical short- to mid-term studies, complications remain a concern. Overall complication rates of 11%–18% have been reported, with glenoid loosening accounting for up to 31% of these cases. The technique and type of graft used influence these outcomes.^8,13,14^ The W-AUG-rTSA seems to surpass BIO-rTSA in terms of lower complication and revision rates, lower operative time, and technical simplicity; nevertheless, BIO-rTSA provides greater flexibility to reconstruct multiplanar deformities and restore the glenoid bone.^7,15^

While several studies have investigated the impact of different parameters of non-augmented rTSA implants on glenoid baseplate fixation over the last decade,^1,16–24^ very few studies have addressed the use of augmented techniques.^6,24,25^ Using polyurethane blocks, Stroud et al.^24^ conducted an experimental study to quantify the initial glenoid fixation of 6 augmented and non-augmented rTSA techniques, including BIO-rTSA, submitted to shear and compressive loads before and after cyclic loading. Significant differences in displacements were observed for the different techniques, with micromotions ranging between 100 and 400 µm. Van de Kleut et al.^25^ performed a 2-year randomized clinical trial to directly compare the implant migration and range of motion between the W-AUG-rTSA and BIO-rTSA techniques, using radio-stereometric analysis. The authors reported no differences in migration between the two augmented techniques. However, the study had important limitations: patients with insufficient bone stock were excluded; only a single augmented baseplate design was used, regardless of the specific glenoid defect characteristics; and the cohort exhibited wide variability in bone loss severity and location. Using finite element (FE) models of nine scapulae with Walch-type B2 or B3 glenoid morphology, Kaur et al.^6^ found that the W-AUG-rTSA produced significantly greater micromotions than the angled BIO-rTSA, suggesting a higher likelihood of the W-AUG-rTSA to inhibit bone ingrowth. However, their findings also had limitations: the angled BIO-rTSA was evaluated considering a single material property for the graft; and both techniques considered 4 peripheral screws, even though it may be unfeasible in the clinical practice to consider more than the superior and inferior screws.^17,26^

Given the limited literature, it remains unclear whether the W-AUG-rTSA and angled BIO-rTSA are equivalent in terms of initial fixation. The aim of this study was to compare the initial fixation of the W-AUG-rTSA and angled BIO-rTSA techniques in a retroverted glenoid by evaluating micromotions at the bone-implant interface using 3D finite element models. Additionally, the influence of graft stiffness and the number of peripheral screws on initial fixation was also investigated. Although an improvement in fixation is generally expected with an increased number of screws, the analysis focused on whether acceptable fixation could be achieved using only two screws and whether one technique performed more favorably under such constraints.

## Materials and methods

### Geometric modeling of rTSA

The geometric models of W-AUG-rTSA and angled BIO-rTSA were developed in SolidWorks (Dassault Systèmes, Waltham, MA, USA) through two main steps: first, designing the W-AUG-rTSA and angled BIO-rTSA implants; and second, virtually creating a retroverted shoulder model and performing the implantation using the manufacturers’ surgical guidelines.^27,28^ These steps are further detailed in the next two sub-sections.

#### W-AUG-rTSA and angled BIO-rTSA implants

The W-AUG-rTSA implant was designed based on the Tornier Perform Reverse Augmented Glenoid (Stryker, Portage, MI, USA)^27^ and the angled BIO-rTSA implant was based on the Tornier Perform Reversed Glenoid.^28^ Both systems are composed of a baseplate, one central screw, and four peripheral screws.

**Figure 1.**
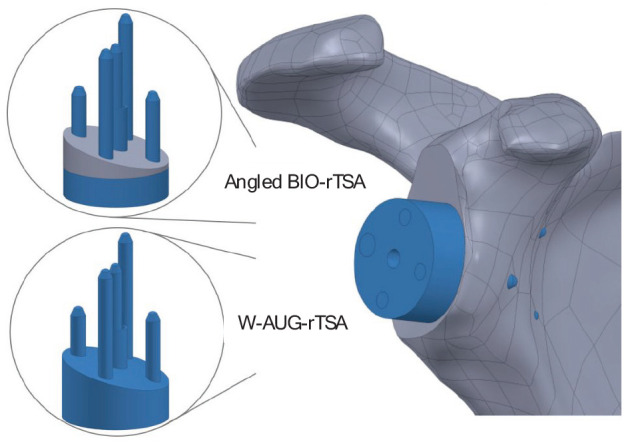
3D geometric models of the angled BIO-rTSA and W-AUG-rTSA implants. The metallic parts are represented in blue and the bone is represented in gray.

In the computational models developed in this study, the W-AUG-rTSA technique included a 15° full-wedge augmented baseplate with a diameter of 25 mm, featuring a wedge portion made of Stryker’s Adaptis Integrated Porous Metal, while the angled BIO-rTSA technique used a non-augmented 25 mm baseplate combined with a trapezoidal bone graft. For each technique, 2 screw configurations were simulated: a 2-screw and a 4-screw setup. In the 2-screw configuration, the anterior and posterior screws were omitted. In the 4-screw configuration, the W-AUG-rTSA technique included a peripheral non-locking screw placed posteriorly, whereas the angled BIO-rTSA technique used peripheral non-locking screws both posteriorly and anteriorly. The remaining positions used locking screws.^27,28^ The screw types used in each technique followed the respective manufacturer’s recommendations. For the sake of computational simplicity, the screws were modeled as cylinders in accordance with the international standards *ISO 5835:1991(en)*^29^ and were positioned parallel to the baseplate axis, as performed in previous FE studies.^5,16,17,19^ In clinical practice, superior screws are typically placed near the base of the coracoid process, and inferior screws are placed along the scapular pillar to optimize bone fixation. The simulated configuration, which assumes a more central screw placement, may therefore represent a worst-case scenario for fixation stability. Non-locking screws were modeled with a head to more accurately represent their interaction with the baseplate. The lengths of the screws were adjusted until bicortical fixation was achieved.^27^ For both models, anterior and posterior peripheral screws were 26 mm long, while superior and inferior peripheral screws were 38 and 42 mm long, respectively (Figure 1).

#### W-AUG-rTSA and angled BIO-rTSA shoulder models

A posteriorly retroverted shoulder model was generated from an intact right scapula model, built from the CT data of the Visible Human Male.^30^ Assuming an idealized bone defect, the retroverted glenoid was simulated using a 3D structure shaped like the rTSA implants. Note that both the W-AUG-rTSA and angled BIO-rTSA implants were assumed to have a similar shape. The instrument was placed distally with respect to the center of the glenoid (specifically, 11.6 mm above the inferior unreamed glenoid rim),^31^ and perpendicular to the glenoid surface plane, until its backside achieved total contact with the glenoid. Subsequently, the 15° retroverted glenoid was generated through a subtractive Boolean operation. Finally, the rTSA implants were positioned in the retroverted shoulder model according to the manufacturer’s recommendations (Figure 1).

### FE modeling of the augmented rTSA techniques

#### Material properties

The 3D rTSA geometric models developed were imported into Abaqus 2017 (Dassault Systèmes, Waltham, MA, USA). All components were modeled as isotropic and linearly elastic. Homogeneous material properties were assigned to the screws, the non-augmented portion of the baseplates, the wedge portion of the W-AUG-rTSA baseplate, and the graft in the angled BIO-rTSA. Heterogeneous material properties were assigned to the scapula based on its bone density distribution.^6,31^ The Young’s modulus and Poisson’s ratio of the screws and the non-augmented baseplate regions were 113.8 GPa and 0.34, respectively.^1,6^ The wedge portion of the W-AUG-rTSA baseplate was assigned a Young’s modulus of 2.5 GPa and a Poisson’s ratio of 0.34.^32,33^

To study the influence of graft stiffness on initial fixation, three Young’s moduli, hereafter referred to as “L-graft,”“H-graft,” and “PM-graft,” were considered: 96 MPa, 1.3 GPa, and 2.5 GPa, respectively. L-graft corresponds to the bone graft stiffness used by Kaur et al.^6^ to facilitate the comparison of results with the literature; H-graft corresponds to the bone graft stiffness of the humeral head with an assumed average bone density of 0.37 g/cm^3^,^34^ estimated using a relationship between Young’s modulus and bone density^35^; and PM-graft corresponds to the Young’s modulus assigned to the Adaptis Integrated Porous Metal of the W-AUG-rTSA baseplate, to allow a more direct comparison of results between the angled BIO-rTSA and W-AUG-rTSA techniques.

Considering a heterogeneous bone density distribution estimated from the CT data, Young’s moduli were assigned individually to each node of the scapula mesh using the relationships between elasticity and bone density proposed by Rice et al.^36^ and Schaffler and Burr,^37^ given by:

(1.a) $$\rho \;<1.54\;\Rightarrow E_{\mathrm{trab}}=60+900\rho^{2}$$

(1.b) $$\rho \geq 1.54\;\Rightarrow E_{\mathrm{cort}}=90\rho^{7.4}$$

where ρ is bone apparent density in g/cm^3^, $E_{\mathrm{trab}}$ is Young’s modulus of trabecular bone in MPa, and $E_{\mathrm{cort}}$ is Young’s modulus of cortical bone in MPa. An in-house Matlab code was used to map the bone densities from the intact scapula to the models of the augmented rTSA techniques on a node-by-node basis.^30^ A Poisson’s ratio of 0.3 was assumed for the scapula. To simulate the outer cortical layer of the scapula, a shell with a thickness of 0.5 mm, a Poisson’s ratio of 0.3, and a Young’s modulus of 17 GPa was added to the FE model.^38^ This layer, hereafter referred to as the cortical shell, did not cover the baseplate region, which consisted of trabecular bone surrounded by cortical bone at the periphery.

#### Interactions

The interactions between the cortical shell and scapula, screws and scapula, and locking screws and baseplate were modeled as fully bonded using tie constraints. Interactions involving non-locking screws and baseplate, baseplate and scapula, baseplate and graft, and graft and scapula were modeled through surface-to-surface contact with friction coefficients of 0.36, 0.74, 0.74, and 0.91, respectively.^6^ A small sliding formulation was selected for all these interactions. In the angled BIO-rTSA model, the interaction between the peripheral screws and the bone graft was modeled through a frictionless surface-to-surface contact formulation.

#### Loading and boundary conditions

Loading conditions were applied in two steps: a pre-load step, where pre-tensioning was applied to non-locking screws; followed by a loading step, where compressive and shear loads were applied.

In the pre-load step, a bolt force of 175 N was applied to the non-locking screws to model the tightening screw force. This bolt force is expected to represent the tightening torque applied by the surgeon. Its magnitude was based on the experimental results of Terrier et al.^39^

In the loading step, compressive and inferior-to-superior shear loads, both with 750 N, were applied at the rTSA joint center to simulate the force that the humeral component exerts on the glenosphere, as performed in previous FE studies,^1,6,10,19^ and in accordance with the *ASTM F2028-17* standard guidelines for testing rTSA implants.^40^

An encastre condition was defined at the medial border of the scapula, where the rhomboideus muscles insert, to prevent rigid body motion.^1,41^

#### Mesh

The scapula was meshed with quadratic tetrahedral (C3D10) elements, since its complex geometry precluded the generation of hexahedral elements, and had an average element edge length of 1.5 mm. The cortical shell was meshed with linear triangular (S3) elements, with an average element edge length of 1.0 mm. Linear hexahedral (C3D8) elements, with an average element edge length of 0.5 mm, were used for the remaining parts. A mesh convergence analysis confirmed that these mesh sizes were appropriate, as further refinement resulted in changes of less than 1% in the predicted maximum micromotion. The selected mesh sizes are also consistent with those adopted in similar FE analyses.^6,20^

### Model validation

The developed FE models were validated by reproducing the displacement test performed experimentally by Stroud et al.^24^ In their study, the baseplate fixation was quantified, before and after cyclic loading, for six rTSA implants, which were implanted in low and high-density polyurethane bone-substitute blocks. Shear loads (applied in the inferior-to-superior and anterior-to-superior directions, alternately) and compressive loads of 357 N and 50 N, respectively, were applied, and the displacement between the polyurethane bone-substitute blocks and rTSA implants was measured in the direction of the applied loads using a dial indicator. Validation was only performed for the angled BIO-rTSA model, as no data were available for the W-AUG-rTSA model. To simulate the high-density polyurethane bone-substitute blocks used in the experimental study, a Young’s modulus of 270 MPa was applied to both the scapula and bone graft. The displacement in the inferior-to-superior direction was assessed at the superior end of the glenoid, aiming to closely replicate the experimental conditions. This computational result was compared to the average displacement obtained with their closest analogous BIO-rTSA model, prior to cyclic loading.

### Evaluation of initial fixation

Micromotions occurring at the bone-implant interface were evaluated for both W-AUG-rTSA and angled BIO-rTSA models, considering grafts with varying Young’s moduli (L-graft, H-graft, and PM-graft) and a different number of peripheral screws (2 vs 4). For simplicity, the term “bone-implant interface” is used for both techniques. However, note that in the W-AUG-rTSA technique, it refers to the contact between the metallic surface of the baseplate – including the central peg – and the glenoid bone, whereas in the angled BIO-rTSA technique, it refers to both the contact between the central peg and the glenoid bone and the contact between the graft surface and the glenoid. Despite material differences, these interfaces are geometrically comparable, making them suitable for direct comparison. Moreover, although micromotions between the metallic surface of the baseplate and the graft in the angled BIO-rTSA technique are not presented in this manuscript, this interface was verified to be less critical than the graft-bone interface, justifying the focus on the latter. For the sake of briefness, the different number of peripheral screws was only assessed considering the H-graft in the angled BIO-rTSA technique.

An in-house Matlab program was used to compute micromotions by subtracting the displacement of an implant node from the displacement of the corresponding bone node. When bone meshes did not match implant meshes at the interface, interpolation was performed to find matching nodes. The computed micromotions included both tangential and normal components at the interface.^38,42,43^ The percentage of nodes with micromotions exceeding a bone ingrowth threshold of 50 µm, was recorded for both techniques, since the threshold for achieving good-quality bone on-growth has been assumed to range from 20 to 50 µm, with fibrous tissues formation more likely to occur between 50 and 150 µm.^17,44^

## Results

### Model validation

The displacement obtained while reproducing the mechanical experiment of Stroud et al.^24^ was 103 µm.

### W-AUG-rTSA versus angled BIO-rTSA: 4 peripheral screw system

**Figure 2.**
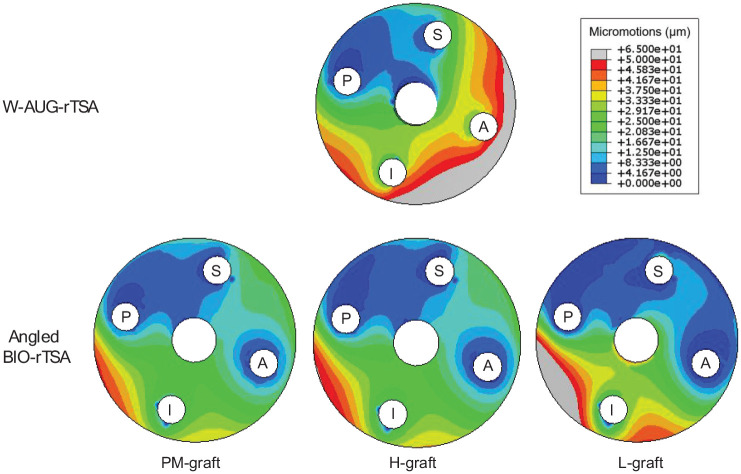
Micromotion distributions (in µm) at the bone-implant interface, for the W-AUG-rTSA and angled BIO-rTSA techniques, using grafts with different Young’s moduli (2.5 GPa, 1.3 GPa, and 96 MPa for PM-graft, H-graft, and L-graft, respectively). Gray regions identify the glenoid regions where the 50 µm bone ingrowth threshold is exceeded.

Figure 2 presents the micromotion distributions at the bone-implant interface for both W-AUG-rTSA and angled BIO-rTSA techniques. The micromotion magnitudes for the W-AUG-rTSA technique ranged from 0 to 63.5 µm, with a median of 24.8 µm. For the angled BIO-rTSA technique, micromotion magnitudes ranged from 0 to 47.4 µm, with a median of 14.9 µm, when considering the PM-graft; from 0 to 50.8 µm, with a median of 14.2 µm, when considering the H-graft; and from 0 to 65.0 µm, with a median of 12.9 µm, when considering the L-graft. In the W-AUG-rTSA technique, 9% of the interface nodes recorded micromotions exceeding 50 µm. In the angled BIO-rTSA, this threshold was exceeded only with the L-graft, and in just 4% of the interface nodes. For the W-AUG-rTSA, the highest micromotions were primarily observed in the inferior and anterior regions of the glenoid surface, whereas for the angled BIO-rTSA, they were predominantly concentrated in the inferior and posterior regions.

### W-AUG-rTSA versus angled BIO-rTSA: 2 versus 4 peripheral screws

**Figure 3.**
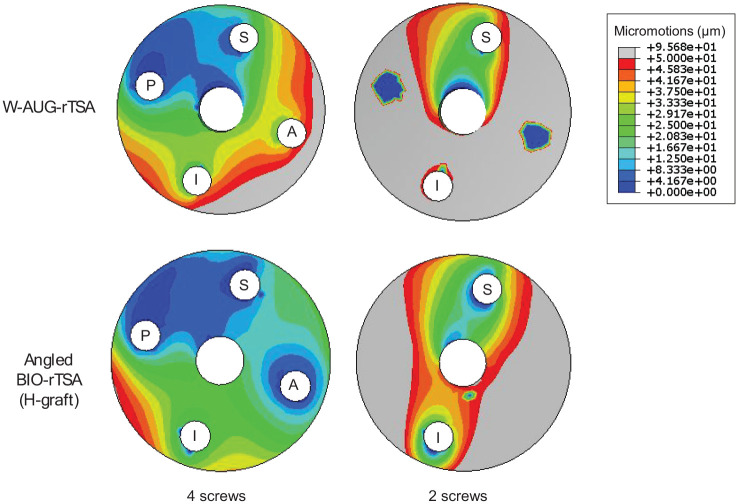
Micromotion distributions (in µm) at the bone-implant interface, for the W-AUG-rTSA and angled BIO-rTSA with the H-graft, using 4 versus 2 peripheral screws. The gray regions identify the regions where the 50 µm bone ingrowth threshold is exceeded.

Figure 3 compares the micromotion distributions (in µm) of both techniques at the bone-implant interface using 2 versus 4 peripheral screws. When only the superior and inferior screws were used, the maximum micromotion increased by 51% (from 63.5 to 95.7 µm) and 60% (from 50.8 to 81.4 µm) in both W-AUG-rTSA and angled BIO-rTSA, respectively. The median micromotion for the W-AUG-rTSA and angled BIO-rTSA using 2 peripheral screws was, respectively, 52.6 and 42.8 µm. The percentage of glenoid surface nodes exceeding the bone ingrowth threshold increased from 9% to 55% in the W-AUG-rTSA and from 0% to 39% in the angled BIO-rTSA (with the H-graft). The greatest micromotions were recorded in the anterior and posterior regions for both techniques, and also in the inferior region in the case of the W-AUG-rTSA. The two circular regions in the W-AUG-rTSA model with zero micromotion correspond to regions where the glenoid does not contact the baseplate, as these regions align with the baseplate holes for the anterior and posterior screws.

## Discussion

The initial fixation of the W-AUG-rTSA and angled BIO-rTSA techniques was investigated in a 15° retroverted glenoid using 3D finite element models. The results suggest that both augmentation techniques can likely achieve good initial fixation when 4 peripheral screws are used. Additionally, although lower graft stiffness led to differences in distribution and generally larger micromotion magnitudes, the angled BIO-rTSA exhibited limited sensitivity to graft stiffness in terms of fixation stability, with only 4% of interface nodes exceeding the 50 μm threshold in the least stiff condition. In contrast, configurations using only two screws – typically placed superiorly and inferiorly – showed markedly poorer fixation in both techniques. The addition of anterior and posterior screws enhanced stability, underscoring the importance of a comprehensive peripheral screw strategy.

The methodology used in this work was validated by reproducing the mechanical experiment of Stroud et al.^24^ using the angled BIO-rTSA model. The authors reported an average displacement of 160 µm in the inferior-to-superior direction for their BIO-rTSA design. In comparison, the displacement obtained in this study was 36% lower, which may be due to differences in testing conditions: polyurethane blocks consist solely of an homogeneous soft material, meaning the screws are anchored in a soft material, whereas in the developed FE models, the screws are anchored in a cortical shell that provides superior structural support; this study used a trapezoidal graft to correct the retroverted glenoid, in contrast to the cylindrical graft used by Stroud et al.^24^; the screws used here have a larger diameter; and the glenoid surface has a narrower surface compared to the polyurethane block, making it unfeasible to measure displacements at the distance considered by Stroud et al.^24^ Additionally, while the experiment was reproduced to the best of the authors’ knowledge, other differences in methodology may exist due to the limited detail available on certain experimental conditions.

Considering 4 peripheral screws, the W-AUG-rTSA technique presented overall higher micromotions at the bone-implant interface compared with the angled BIO-rTSA technique. These findings align with those reported by Kaur et al.,^6^ who directly compared the micromotions at the bone-implant interface with these 2 augmented techniques, using 9 scapulae FE models with Walch-type B2 or B3 glenoid morphologies. In their study, both implants were fixed using a central peg and 4 peripheral screws, and the graft used in the angled BIO-rTSA had a stiffness of 96 MPa, like L-graft used here.

Micromotions at the bone-implant interface are often suggested to have to remain below 50 µm for adequate bone ingrowth to occur.^17,44^ In this study, approximately 9% and 4% of the glenoid surface exceeded this threshold in the W-AUG-rTSA and angled BIO-rTSA with the L-graft, respectively, when 4 peripheral screws were used. Since only a small percentage of the surface exceeded the bone ingrowth threshold in both techniques, and in the case of the angled BIO-rTSA, this only occurred under conditions of graft stiffness that likely represent a worst-case scenario, these results suggest that both techniques likely achieve good initial fixation. Furthermore, the initial fixation in the angled BIO-rTSA may exhibit limited sensitivity to the bone graft stiffness.

The largest micromotions in both techniques were observed at the inferior region of the glenoid due to the moment created by the inferior-to-superior shear force (whose direction is aligned with the superior and inferior screws). The increased micromotions observed at the anterior region of the glenoid for the W-AUG-rTSA result from the type of anterior screw used. Unlike the W-AUG-rTSA, which uses an anterior locking screw, the angled BIO-rTSA uses a non-locking screw. This non-locking configuration likely confers an additional compression at the bone-implant interface, decreasing micromotions.

Although most augmented baseplates accommodate up to 4 screws, particular scenarios may arise in the clinical practice where only the superior and inferior screws can be applied.^17,26^ This limitation occurs because additional screws can diminish the available bone stock for baseplate support, and optimal screw placement for adequate initial fixation may not always be feasible^22^ The number of peripheral screws necessary to achieve good initial fixation has been studied previously in non-augmented baseplates with controversial conclusions. James et al.^22^ found no differences in micromotions between using 2 versus 4 peripheral screws, suggesting that the anterior and posterior screws contribute little to the fixation of the baseplate. In contrast, Hoenig et al.^23^ concluded that the posterior screw significantly enhances the glenoid component stability, especially in patients with bone loss or defects. To the author’s knowledge, no studies have specifically investigated the impact of the number of screws on the initial fixation of augmented rTSA techniques. In the current study, the reduction from 4 to 2 screws substantially increased the percentage of interface nodes with micromotions above the 50 µm threshold in both the W-AUG-rTSA and angled BIO-rTSA with the H-graft, suggesting that the anterior and posterior screws play an important part in enhancing baseplate fixation.

The findings of this study should be interpreted taking into consideration its inherent limitations. First, the computational models applied were developed from the CT images of a single healthy individual, whose retroversion was created virtually. Consequently, the results do not encompass the anatomical and morphological variations in scapular geometry or density distribution within the population submitted to rTSA. Second, the relationships between bone density and elasticity used to assign non-homogeneous material properties for the scapula were not scapula-specific. However, they are likely to have a limited effect on the qualitative findings of this study, as demonstrated by Kusins et al.^31^ Third, the screws were modeled as non-threaded cylinders, as performed in several FE studies.^16,17,19^ Fourth, all screws were placed parallel to the baseplate axis, as previously performed by Bonnevialle et al.^20^ As placing the screws in a divergent configuration would offer increased stability, the results presented in this study likely represent the worst-case scenario. Fifth, even though the outcomes of this study are aligned with the experimental and computational studies of Stroud et al.^24^ and Kaur et al.,^6^ respectively, strengthening the confidence in them, model validation was only conducted indirectly. Sixth, only one threshold was considered to compare the techniques. When a more conservative threshold of 20 µm^17,44^ was applied, the percentage of nodes exceeding this value increased substantially, reaching 58% and 32%–41% for the W-AUG-rTSA and angled BIO-rTSA techniques, respectively, with 4 screws. These findings suggest that under stricter criteria for bone ingrowth, the initial fixation of both techniques is more limited. Conversely, when a more relaxed threshold of 150 µm^45^ was applied, both techniques exhibited good initial fixation, as no node exceeded this threshold, even when only 2 screws were used. Finally, the comparison performed between techniques was purely biomechanical and focused on initial fixation only. Other parameters, such as graft resorption in the angled BIO-rTSA, may influence the overall outcome of the techniques but were not considered in this study. From a clinical perspective, other factors should also be considered in the decision-making process regarding the most suitable option for a given patient, since the techniques have different degrees of execution difficulty, surgical time, cost-effectiveness, flexibility, complication, and revision rates.^9,15,19^

## Conclusions

This 3D finite element study compared the initial fixation of the metallic wedge-augmented (W-AUG-rTSA) and angled bony increased-offset (angled BIO-rTSA) reverse total shoulder arthroplasty techniques in a 15° retroverted glenoid. Overall, the results suggest that both augmentation techniques can likely achieve good initial fixation when 4 peripheral screws are used. Reducing the number of screws from 4 to 2 notably affected their initial fixation. Additionally, variations in graft stiffness had a limited influence on initial fixation in the angled BIO-rTSA, despite some differences in micromotion distribution.
